# Gene crosstalk between COVID-19 and preeclampsia revealed by blood transcriptome analysis

**DOI:** 10.3389/fimmu.2023.1243450

**Published:** 2024-01-08

**Authors:** Yijing Chu, Min Li, Mingze Sun, Jing Wang, Wan Xin, Lin Xu

**Affiliations:** Department of Obstetrics, the Affiliated Hospital of Qingdao University, Qingdao, China

**Keywords:** COVID-19, preeclampsia, differentially expressed genes, protein-protein interaction, drug molecule

## Abstract

**Background:**

The extensive spread of coronavirus disease 2019 (COVID-19) has led to a rapid increase in global mortality. Preeclampsia is a commonly observed pregnancy ailment characterized by high maternal morbidity and mortality rates, in addition to the restriction of fetal growth within the uterine environment. Pregnant individuals afflicted with vascular disorders, including preeclampsia, exhibit an increased susceptibility to severe acute respiratory syndrome coronavirus 2 (SARS-CoV-2) infection via mechanisms that have not been fully delineated. Additionally, the intricate molecular mechanisms underlying preeclampsia and COVID-19 have not been fully elucidated. This study aimed to discern commonalities in gene expression, regulators, and pathways shared between COVID-19 and preeclampsia. The objective was to uncover potential insights that could contribute to novel treatment strategies for both COVID-19 and preeclampsia.

**Method:**

Transcriptomic datasets for COVID-19 peripheral blood (GSE152418) and preeclampsia blood (GSE48424) were initially sourced from the Gene Expression Omnibus (GEO) database. Subsequent to that, we conducted a subanalysis by selecting females from the GSE152418 dataset and employed the “Deseq2” package to identify genes that exhibited differential expression. Simultaneously, the “limma” package was applied to identify differentially expressed genes (DEGs) in the preeclampsia dataset (GSE48424). Following that, an intersection analysis was conducted to identify the common DEGs obtained from both the COVID-19 and preeclampsia datasets. The identified shared DEGs were subsequently utilized for functional enrichment analysis, transcription factor (TF) and microRNAs (miRNA) prediction, pathway analysis, and identification of potential candidate drugs. Finally, to validate the bioinformatics findings, we collected peripheral blood mononuclear cell (PBMC) samples from healthy individuals, COVID-19 patients, and Preeclampsia patients. The abundance of the top 10 Hub genes in both diseases was assessed using real-time quantitative polymerase chain reaction (RT-qPCR).

**Result:**

A total of 355 overlapping DEGs were identified in both preeclampsia and COVID-19 datasets. Subsequent ontological analysis, encompassing Gene Ontology (GO) functional assessment and Kyoto Encyclopedia of Genes and Genomes (KEGG) pathway analysis, revealed a significant association between the two conditions. Protein-protein interactions (PPIs) were constructed using the STRING database. Additionally, the top 10 hub genes (MRPL11, MRPS12, UQCRH, ATP5I, UQCRQ, ATP5D, COX6B1, ATP5O, ATP5H, NDUFA6) were selected based on their ranking scores using the degree algorithm, which considered the shared DEGs. Moreover, transcription factor-gene interactions, protein-drug interactions, co-regulatory networks of DEGs and miRNAs, and protein-drug interactions involving the shared DEGs were also identified in the datasets. Finally, RT-PCR results confirmed that 10 hub genes do exhibit distinct expression profiles in the two diseases.

**Conclusion:**

This study successfully identified overlapping DEGs, functional pathways, and regulatory elements between COVID-19 and preeclampsia. The findings provide valuable insights into the shared molecular mechanisms and potential therapeutic targets for both diseases. The validation through RT-qPCR further supports the distinct expression profiles of the identified hub genes in COVID-19 and preeclampsia, emphasizing their potential roles as biomarkers or therapeutic targets in these conditions.

## Introduction

The global outbreak of COVID-19 can be attributed to the emergence of the SARS-CoV-2 virus, and has caused unprecedented health consequences across the world (1). SARS-CoV-2 infection occurs through exposure to respiratory aerosols and droplets (2–4). The virus is capable of inducing direct endothelial injury, thrombo-inflammation, immune system dysregulation, and changes in ACE2-associated pathways (5–8). Severe COVID-19 is typically characterized by pulmonary infection accompanied with cough, fever and dyspnoea (9). The most severe pathophysiologic symptoms of COVID-19 include the damage of pulmonary epithelia, hypercoagulation, thrombosis and excessive vascular permeability resulting into sepsis (10).

Preeclampsia, an ailment associated with pregnancy that commonly presents around the 20-week gestation mark, and is symptomized by placental oxidative stress, endothelial damage and antiangiogenesis that induce proteinuria, hypertension, and similar multiorgan responses as observed in severe COVID-19 cases (11–14). It has been reported in recent studies that there is a strong connection of COVID-19 with preeclampsia in pregnant patients (15, 16). The infection caused by SARS-CoV-2 during pregnancies can induce risks for both the mother and the developing fetus, contributing to pregnancy-related complications like preeclampsia and impaired intrauterine growth (17–26). Several studies have indicated an elevated likelihood of perinatal outcomes in pregnant individuals with COVID-19, including more proneness to preeclampsia and premature parturition (27). It is thus important to assess the impact of COVID-19 on the preeclampsia patients, and identify potential therapeutic interventions to lower the likelihood of hospitalization or mortality.

Through the analysis of blood RNA sequencing data from individuals with COVID-19 and preeclampsia, this study reveals shared DEGs. Moreover, to validate our bioinformatics findings, we performed experimental validation using PBMC samples obtained from healthy individuals, COVID-19 patients, and preeclampsia patients. Our findings further reinforce the potential of the identified molecules in serving as therapeutic targets for both COVID-19 and preeclampsia, providing valuable insights for the development of effective treatments.

## Materials and methods

### Data source

Within the GEO database (https://www.ncbi.nlm.nih.gov/geo/), we accessed the expression profiles for this study. The COVID-19 datasets (GSE152418) involved RNA-Seq profiling of peripheral blood samples from 17 COVID-19 patients and 17 healthy subjects (28). Furthermore, the preeclampsia dataset (GSE48424) consisted of whole-blood RNA-seq data from 18 preeclampsia patients and 18 healthy individuals (29).

### Identification of common DEGs in GSE152418 and GSE48424

First, female patients were selected from the GSE48424, including 9 COVID-19 patients and 9 healthy subjects. To identify DEGs in the GSE152418 dataset, we utilized the R package “Deseq2” and applied a significance threshold of padj = 0.05 and |log2 Fold-Change| = 0.5 (30). Additionally, for the GSE48424 dataset, we employed the R package “limma” and identified DEGs using criteria of |log2 Fold-Change| = 0.3 and P-value = 0.05 (31).

### Functional analysis of DEGs

Through GO enrichment and KEGG pathway analyses conducted with the “clusterProfiler” R package, we determined the noteworthy functions and pathways linked to the DEGs, considering a threshold of P-value = 0.05 (32).

### Integration and analysis of protein-protein interactions

The STRING database (www.string-db.org) was employed to analyze and integrate the DEGs, resulting in the construction of a PPI network with a median confidence score of 0.7 (33). Subsequently, Cytoscape software was utilized for visualization and subsequent analysis of the network (34).

### Extraction of hub genes

CytoHubba, an essential plug-in embedded within Cytoscape, provides a comprehensive platform for assessing and identifying the influential modulators of biological networks, harnessing the potential of network metrics (35). CytoHubba was instrumental in examining and scrutinizing the significant nodes present in the modules of the PPI network, thereby uncovering the hub genes (36). By employing the Degree algorithm, the ten genes demonstrating the greatest significance were identified, and their rankings were visually illustrated in plots featuring a gradual change in colors from purple to pink. The hub genes were systematically ranked according to the shortest accessible paths connecting them, facilitating a more accessible and comprehensible visualization of their interrelationships.

### Unraveling of common DEGs-associated transcription factors and miRNAs

Transcription factors (TFs) exert control over transcription and chromatin structure by binding to specific DNA sequences. They form a complex expression-control system within the genome, and is thus crucial for the understanding of involved molecular mechanisms (37). Enrichr operates as a robust gene set search platform that consolidates a broad range of biological information for advanced analysis (38). Common DEGs were imported into Enrichr to infer complete chart of TFs. The top 10 TFs were then selected according to their composite scores. MiRTarBase was used for identifying miRNAs that could interact with the common DEGs via the network analysis tool (39). In addition, the miRTarBase component within Enrichr was performed to explore gene-miRNA connections. Finally, Cytoscape was implemented to exhibit TF-gene and miRNA-gene interactions.

### Associations between gene and disease

DisGeNET (http://www.disgenet.org/) is a comprehensive database that connects genes to human disorders (40). DisGeNET was used to perform gene-disease correlation analysis and visualization using Cytoscape.

### Exploring pharmaceutical agents for COVID-19 and preeclampsia

The analysis encompassed an assessment of promising pharmaceutical agents for the management of COVID-19 and preeclampsia. DSigDB is a novel gene-set repository that connect drugs/medicines to target genes for further enrichment analysis (41). A meticulous evaluation resulted in the identification of a set of 10 highly promising candidates, paving the way for subsequent analytical investigations. PubChem, accessible at (https://pubchem.ncbi.nlm.nih.gov), serves as a comprehensive repository housing extensive data regarding chemical compounds and their associated biological functions, enabling the collaborative exchange, examination, and integration of information from diverse databases. Molecular compositions and two-dimensional arrangements of prospective medications were obtained from PubChem to facilitate pharmaceutical research.

### RT-qPCR

We collected peripheral blood cell samples obtained from four distinct groups, including 30 individuals in each group: healthy individuals, COVID-19 patients, Preeclampsia patients, and pregnant women without any complications. Total RNA was extracted from the peripheral blood cell samples using TRIzol reagent (Vazyme, Cat: R401-01). Subsequently, reverse transcription was performed to synthesize complementary DNA (cDNA) using a reverse transcription kit (Vazyme, Cat: R211-01). RT-PCR was then employed to quantitatively measure the expression levels of the top 10 hub genes identified from the bioinformatics analysis. To ensure data normalization, the expression levels of target genes were normalized against GAPDH as an internal reference. The sequence of primers are shown in **Supplementary Table 1**.

### Statistical analysis

In this study, transcriptomic datasets for COVID-19 and preeclampsia were acquired from the Gene Expression Omnibus database. Utilizing the “Deseq2” and “limma” packages, differential gene expression analysis identified significant genes with adjusted p-values < 0.05. An intersection analysis revealed common differentially expressed genes through hypergeometric testing. Functional enrichment analysis, including Gene Ontology and Kyoto Encyclopedia of Genes and Genomes pathways, was performed on identified genes. Protein-protein interaction networks were constructed using the STRING database, and the top 10 hub genes were determined based on degree algorithm ranking scores. Experimental validation through RT-qPCR on peripheral blood mononuclear cell samples from healthy individuals, COVID-19 patients, and preeclampsia patients confirmed distinct gene expression profiles. The expression levels of the specified genes were evaluated through RT–qPCR, and the data are reported as the mean ± standard error of the mean (s.e.m.). Statistical significance was determined using a one-way analysis of variance (ANOVA), with * denoting p < 0.05, ** indicating p < 0.01, and *** representing p < 0.001.

## Result

### Uncovering Overlapping DEGs between preeclampsia and COVID-19

**Figure 1:**
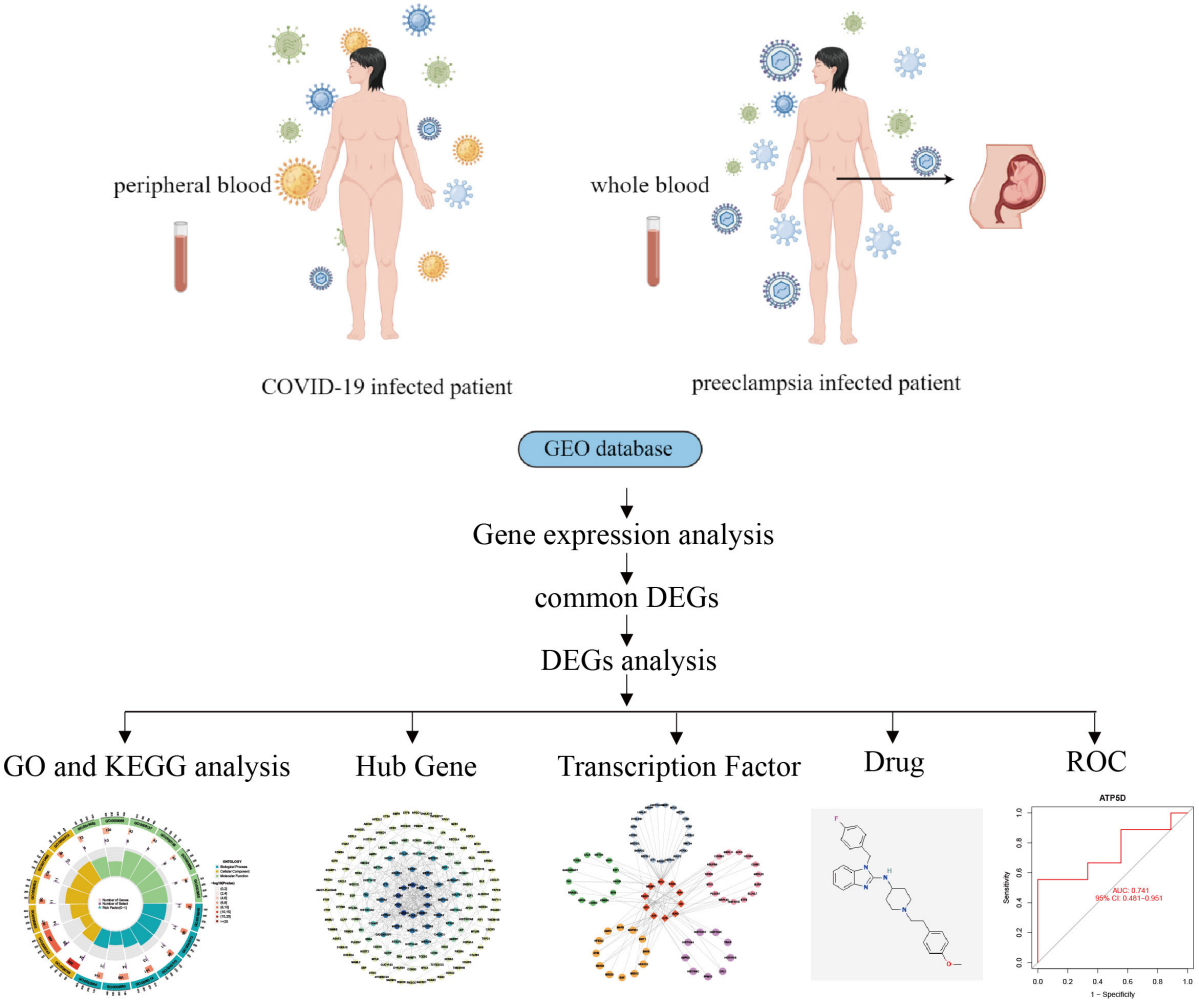
General workflow diagram of this study.

**Figure 2:**
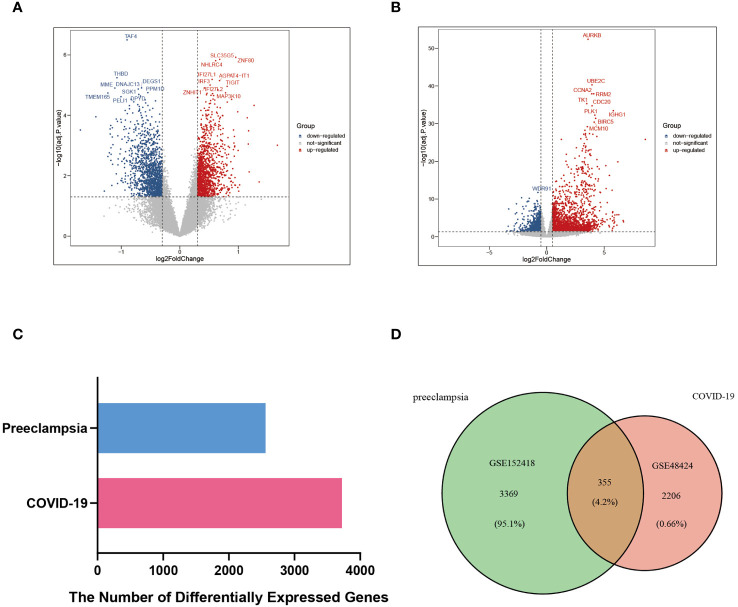
DEGs and common DEGs of the two datasets were visualized between COVID-19 (GSE152418) and preeclampsia (GSE48424). **(A)** Volcano plot of DEGs in the preeclampsia database (GSE48424). Blue represents the downregulated genes, red represents the upregulated genes, and gray represents the rest of DEGs. **(B)** Volcano plot of DEGs in COVID-19 database (GSE152418). Blue represents the downregulated genes, red represents the upregulated genes, and gray represents the rest of DEGs. **(C)** Comparison of the number of DEGs between COVID-19 and preeclampsia. **(D)** Venn diagram visualizing the number of common DEGs between COVID-19 and Preeclampsia.

The critical procedures executed in this research were shown in the flowchart (**Figure 1**). The peripheral blood expression profile was downloaded from GEO, and the genetic elements that could initiate COVID-19 and preeclampsia were identified to examine the interconnectedness between preeclampsia and COVID-19 as well as their effects. First, an amalgamation of 3724 DEGs associated with COVID-19 was identified, consisting of 2682 upregulated genes and 1042 downregulated genes (**Supplementary Table 2**). Within the scope of COVID-19, a combined total of 3724 DEGs was identified, consisting of 2682 genes showing upregulated expression and 1042 genes displaying downregulated expression (**Supplementary Table 3**). The integration of DEGs from both datasets led to the identification of 355 DEGs that were concordantly present (**Figure 2**; **Supplementary Table 4**). The results demonstrated a plausible convergence between the fundamental processes of preeclampsia and COVID-19.

### Investigation of pathway enrichment by analyzing functional annotations

**Figure 3:**
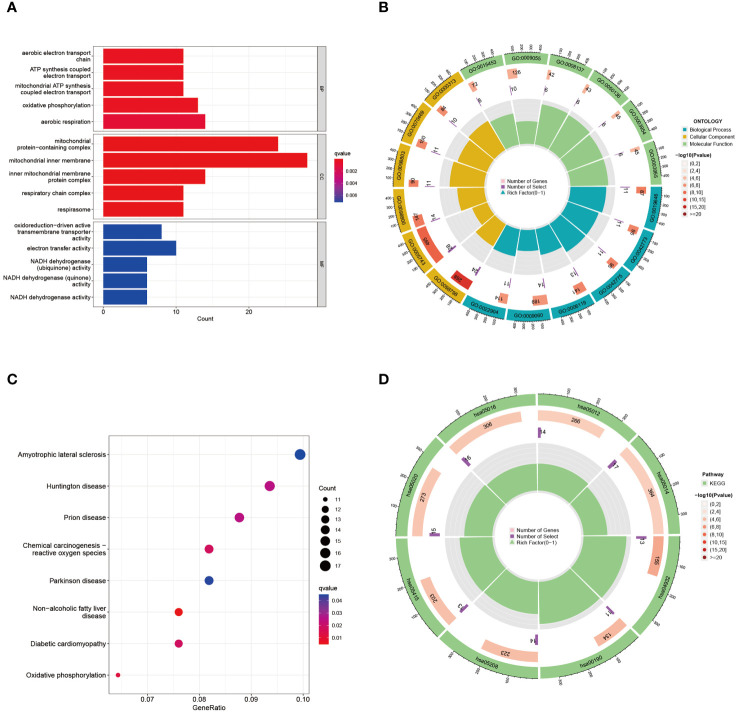
GO and KEGG functional enrichment analysis of the common DEGs between COVID-19 and preeclampsia. **(A)** GO enrichment analysis histogram. BP, biological process; CC, cellular components; MF, molecular function. **(B)** Circle diagram of common DEGs from GO enrichment analysis. The ID of the pathway is indicated on the outermost layer, and P values and gene enrichment numbers are indicated on the innermost layer. **(C)** Bubble graph for KEGG enrichment in differential genes. The larger bubble represents enrichment of more genes, and the increasing depth of red color represents more significant differences. **(D)** Circle diagram of common DEGs from KEGG enrichment analysis.

To elucidate the underlying biological functions and enriched pathways linked to these intersecting DEGs, assessments were executed utilizing the “clusterProfiler” algorithm (**Figure 3A**). The GO analysis demonstrated that “aerobic electron transport chain”, “ATP synthesis coupled electron transport”, “mitochondrial ATP synthesis coupled electron transport”, “oxidative phosphorylation”, and “aerobic respiration” in biological process, “mitochondrial protein-containing complex”, “mitochondrial inner membrane”, “inner mitochondrial membrane protein complex”, “respiratory chain complex”, and “respirasome” in cellular component, “oxidoreduction-driven active transmembrane transporter activity”, “electron transfer activity” in molecular function were most significantly involved in the effects of DEGs approach between preeclampsia and COVID-19. The circular representation of enrichment results featured the outer layer denoting the notably enriched pathways, and the inner layer provided details regarding the statistical significance and abundance of genes demonstrating enrichment (**Figure 3B**). The KEGG pathway analysis can be used to analyze the mutual interactions between different diseases via key biological or molecular processes. It was revealed in this study that “ATP metabolic process”, “energy derivation by oxidation of organic compounds”, “aerobic respiration”, “cellular respiration”, and “positive regulation of ion transport” in shaping the pathogenic mechanisms shared by preeclampsia and COVID-19 (**Figure 3C**). Within the circular visualization of KEGG enrichment analysis, the outer layer portrayed the notably enriched pathway, while the innermost layer denoted the P value and quantity of enriched genes (**Figure 3D**). The circular visualizations underscored the significance of enriched pathways and detailed the statistical significance and gene abundance, offering a comprehensive understanding of the molecular underpinnings connecting these two conditions.

### Identification of hub genes

**Figure 4:**
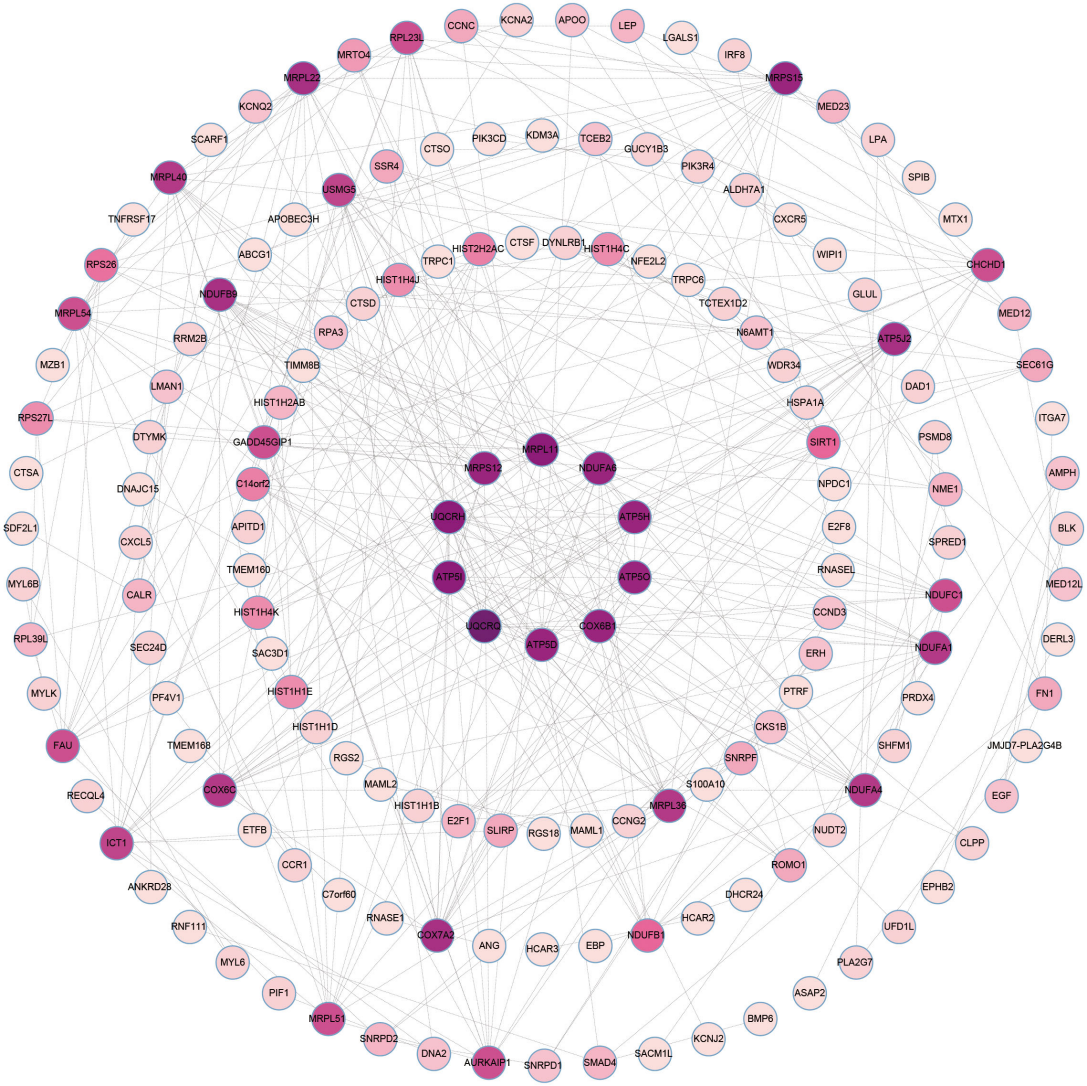
The PPI network of common DEGs between COVID-19 and preeclampsia. The circular node represents DEGs and the edge represents the interaction between nodes.

**Figure 5:**
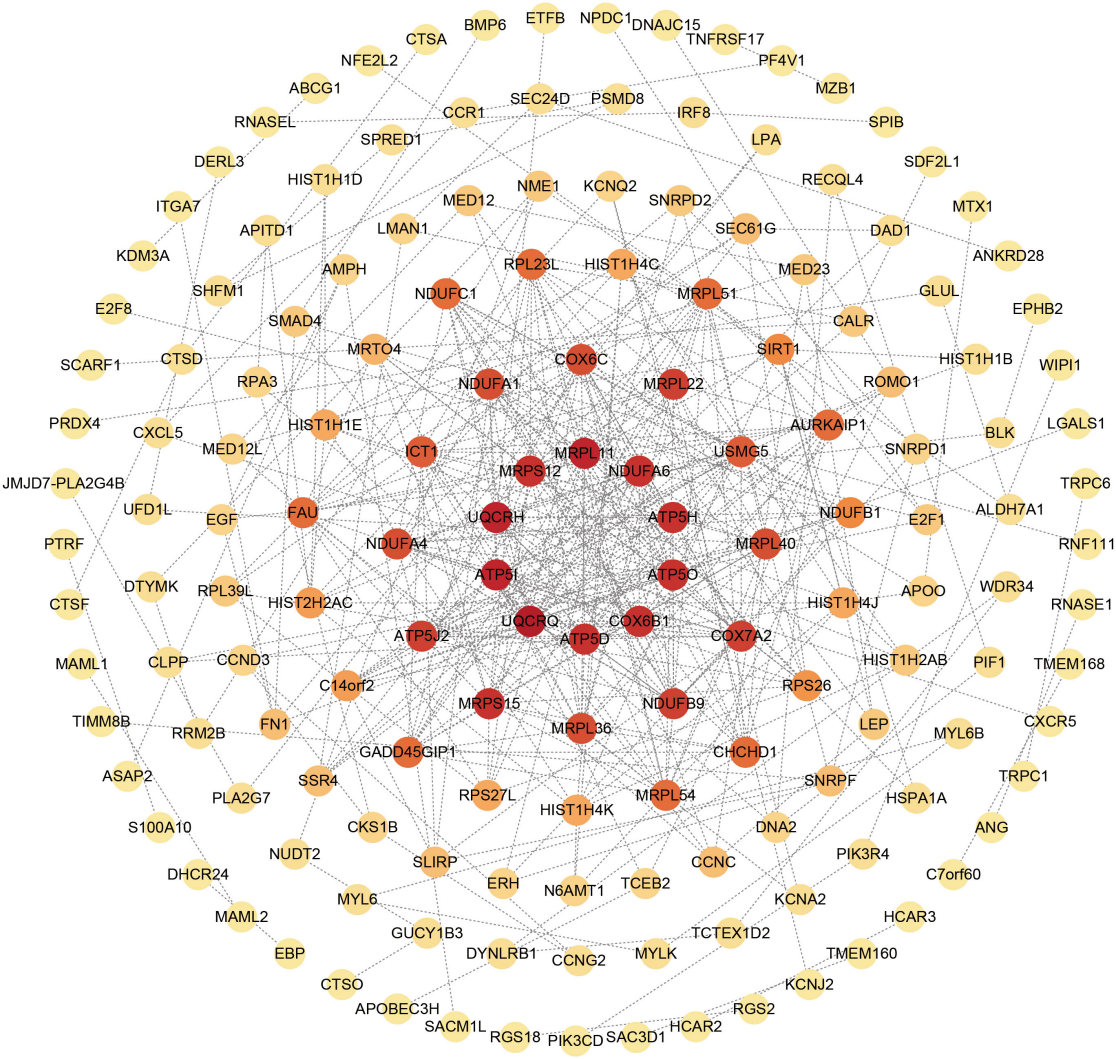
Top 10 hub genes in the PPI network identified based on their scores. In the circle, red stands for the identified top 10 hub genes.

The overlapping DEGs from COVID-19 and preeclampsia datasets were subjected to STRING analysis to reveal protein-protein interactions (PPIs) and determine hub genes (**Figure 4**). By utilizing the Degree algorithm, the top ten genes of remarkable significance were unveiled, assuming pivotal roles as central nodes with exceptional prominence and regulatory functions. These genes include MRPL11, MRPS12, UQCRH, ATP5I, UQCRQ, ATP5D, COX6B1, ATP5O, ATP5H, and NDUFA6 (**Figure 5**). Notably, among these genes, MRPL11 stands out with the highest node count, hinting at its potential to play a pivotal role, given its extensive connectivity within the network. The diagnostic utility of the identified hub genes was examined by conducting ROC analysis for preeclampsia and COVID-19. The AUC values of almost all hub genes were above 0.7 in both pre-eclampsia and COVID-19, highlighting their exceptional prognostic potential in both disease cohorts (**Supplementary Figures 1**, **2**). The findings shed light on the shared genetic mechanisms and offer insights for potential therapeutic strategies in these two diseases.

### Construction of TFs and miRNA regulatory network

**Figure 6:**
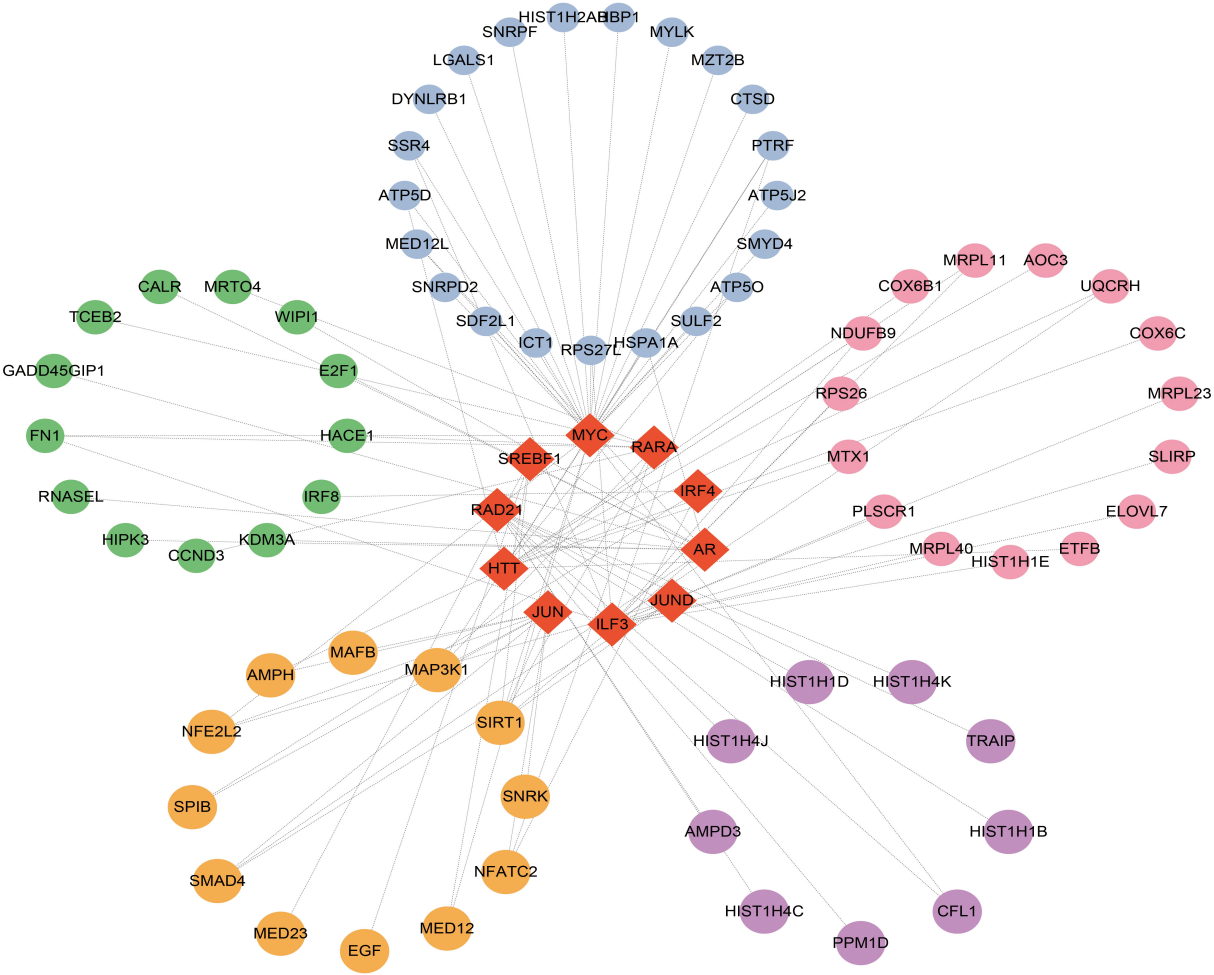
Top 10 transcription factors (TFs) ranked according to P values and their interactions with common DEGs. In this network, the red diamonds represent the top 10TFs with lowest P values. Other colored circles represent common DEGs correlated with TFs.

**Figure 7:**
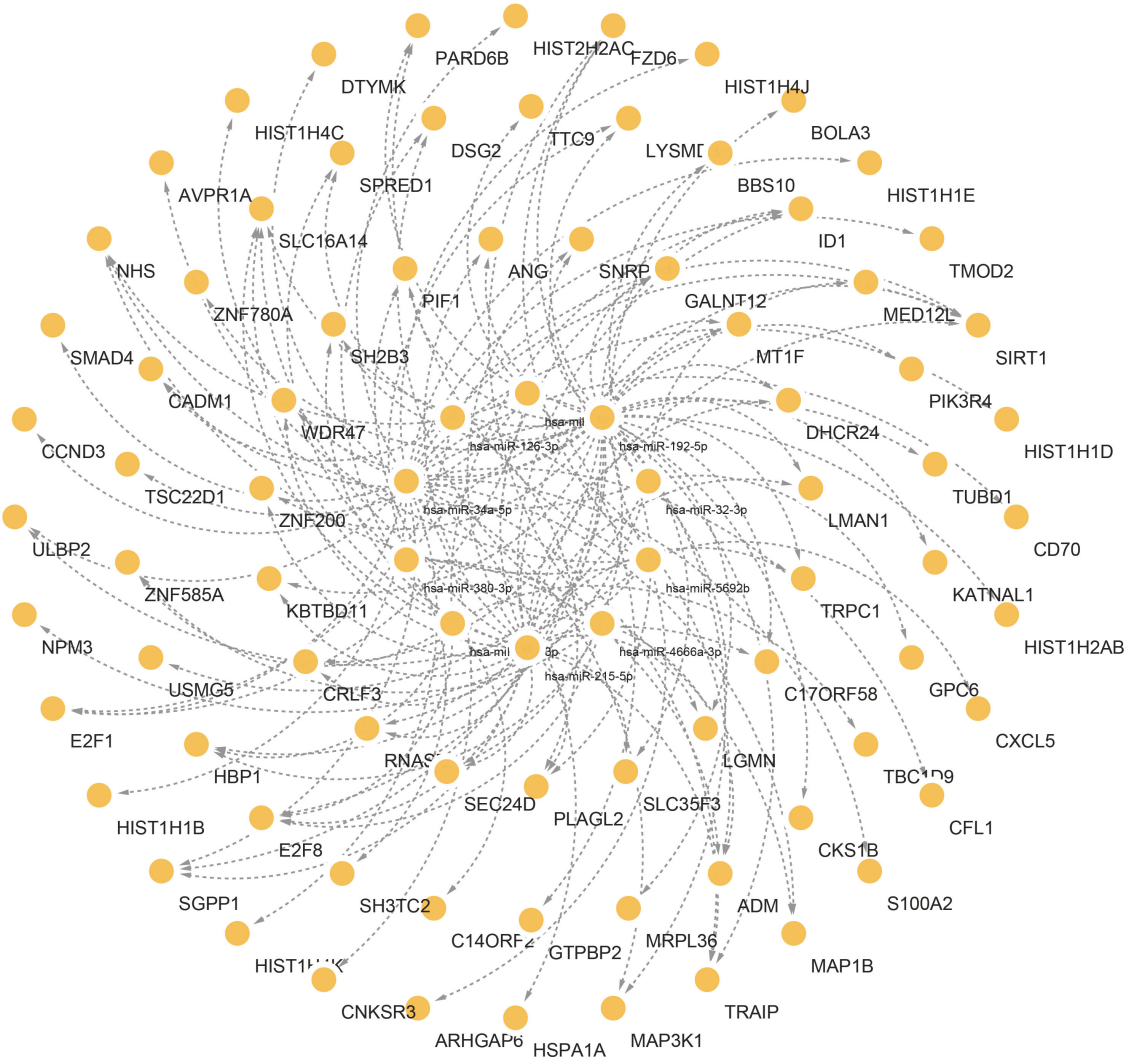
Interconnected regulatory interaction network of the top 10 microRNAs with lowest P values. In the middle circle, the orange circles represent common DEGs correlated with miRNAs.

To identify major transcriptional variations and better understand critical regulatory molecules related to shared DEGs, a web-dependent methodology was utilized to elucidate the governing TFs and miRNAs. The interaction of regulatory TFs and regulatory miRNA factors with common DEGs was respectively displayed in **Figures 6** and **7**. Our results indicated the existence of potential connections between common DEGs and TFs or miRNA (**Supplementary Tables 5**, **6**). These results facilitate further investigations into the specific roles and functions of TFs and miRNAs in the context of the shared DEGs, ultimately advancing our understanding of the biological mechanisms and potential therapeutic targets in the studied diseases.

### Identification of potential drug candidates for both disease

In the pursuit of identifying potential drugs targeting shared DEGs in preeclampsia and COVID-19, a screening process utilizing Enrichr was employed. Transcriptional profiles sourced from DSigDB were utilized to discern ten promising molecules. The selection of the top ten compounds was based on their P-values, indicative of their potential therapeutic significance. **Supplementary Figure 3** showcases the names, chemical formulas, and 2D structures of these drugs. Comprehensive results of all Drug Candidates can be found in **Supplementary Table 7**. These results provided valuable insights and resources for further exploration and potential drug development in the context of these diseases.

### Visual representation of gene-disease associations

Overlapping genetic factors contribute to the interconnectedness of different diseases (42). Upon uploading the shared DEGs of COVID-19 and preeclampsia to DisGeNET for analysis, potential connections emerged between these diseases and various conditions, such as Renal Fibrosis, Ureteral Obstruction, Colonic Neoplasms, Idiopathic Nephrotic Syndrome, IgA Glomerulonephritis, Burkitt Lymphoma, Medullary Neoplasms, Neuroblastoma, Congenital Aneurysm of Ascending Aorta, and Mitochondrial Diseases (**Supplementary Figure 4**, **Supplementary Table 8**). These findings highlight the interplay and shared genetic factors between preeclampsia, COVID-19, and other related diseases, providing valuable insights for further research and a deeper understanding of their pathogenesis and potential therapeutic strategies.

### Validation of hub genes as potential biomarkers in COVID-19 and preeclampsia

**Figure 8:**
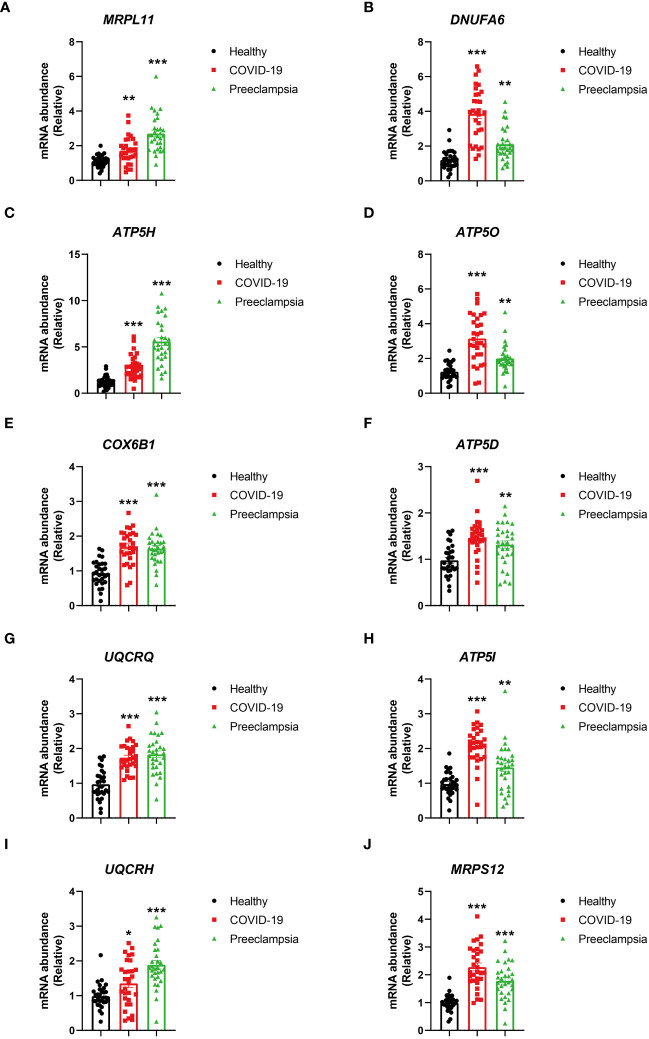
Comparison of HUB gene expression levels in PBMCs from healthy individuals, patients with COVID-19, and patients with Preeclampsia. Expressions of MRPL1 **(A)**, DNUFA6 **(B)**, ATP5H **(C)**, ATP5O **(D)**, COX6B1 **(E)**, ATP5D **(F)**, UQCRQ **(G)**, ATP5I **(H)**, UQCRH **(I)** and MRPS12 **(J)** in healthy individuals, patients with COVID-19, and patients with Preeclampsia. The bar graph depicts the expression levels of HUB genes, with black bars representing healthy individuals, red bars representing COVID-19 patients, and green bars representing Preeclampsia patients. Error bars indicate the standard error of the mean. One-way ANOVA was employed for statistical analysis, and each group comprised 30 individuals. The asterisks on the bar graph indicate the significance of the differences between each group and the healthy group. Significance levels are denoted as follows: p < 0.05, *; p < 0.01, **; p < 0.001, ***.

To establish the credibility of hub genes as potential biomarkers in COVID-19 and Preeclampsia, PBMC were gathered from four distinct populations, including female patients with COVID-19, healthy females, patients with Preeclampsia, and healthy pregnant women. The RT-PCR analysis of the PBMC samples revealed significant differences in mRNA abundance of the hub genes (MRPL11, MRPS12, UQCRH, ATP5I, UQCRQ, ATP5D, COX6B1, ATP5O, ATP5H, and NDUFA6) between COVID-19 and Preeclampsia cohorts, respectively (**Figure 8**). Consistent with the bioinformatics analysis, these hub genes exhibited distinct expression patterns. Notably, PBMC samples from healthy individuals displayed differential expression profiles compared to patients diagnosed with COVID-19 and Preeclampsia, suggesting the prospective applicability of the genes as biomarkers for assessing the presence and progression of both conditions. The distinct expression patterns of these hub genes align with the bioinformatics analysis, suggesting their prospective applicability as reliable biomarkers for assessing the presence and progression of both conditions, providing valuable insights for diagnostic and monitoring purposes.

## Discussion

It has been demonstrated by more and more recent studies that plausible associations could exist between distinct medical conditions. Identification of these associations can be a promising step for future disease studies (43–45). Public health systems are facing unprecedented challenges due to the COVID-19 pandemic. In spite of being primarily deemed as a respiratory complication, infection with SARS-CoV-2 has the potential to influence on endothelial cells, resulting into microvascular dysfunction, microthrombosis and endotheliitis (46). It has been shown recently that preeclampsia patients are more prone to SARS-CoV-2 infection and high COVID-19 mortality than healthy populations (18, 47–49). Instead of immunosuppression observed during normal pregnancies, preeclampsia patients shows aberrant and excessive activation of immune system, characterized by an upregulation of antiangiogenic factors and proinflammatory cytokines in both the maternal endothelia and intrauterine environment, inducing placental insufficiency and systemic complication (50, 51). However, it is still unclear what molecular mechanism contributes to a worse COVID-19 prognosis among preeclampsia patients. Inspired by the finding of possible correlation of Coronavirus infection and an increased risk of preeclampsia during gestation, we investigated the possible connections between preeclampsia and COVID-19 at the transcriptomic level. A network-centric strategy was utilized for analyzing the RNA-seq profiling datasets for both preeclampsia and COVID-19, and for identifying the potential diagnostic biomarkers for preeclampsia among COVID-19 pregnancies.

MRPL11 is a coding gene, and has connection with diseases such as Dyskinetic Cerebral Palsy (52). MRPS12 is located cytogenetically on 19q13.2, and responsible for encoding a 28S ribosomal subunit of S12P family. It has been reported in previous studies that MRPS12 is a critical component of small ribosomal subunits, and regulates the fidelity of decoding and the vulnerability to aminoglycosidic antibiotics (53, 54). An upregulation of MRPS12 is observed in different types of cancer as opposed to non-tumor controls (55). UQCRH is a vital element of the multisubunit transmembrane complex within the mitochondrial electron transport chain, facilitating the movement of electrons from cytochrome c to c1 (56). UQCRH can serve as an indicative marker for assessing the prognosis of liver cancer. Alteration in mitochondrial respiration could be conducive to tumorigenic processes through aberrant biological stress, such as reactive oxygen species (57). ATP5I assists in forming the structure of ATP-generating enzyme, correlating with oxidative phosphorylation in the mitochondria (58). UQCRQ mutation can lead to deficiency of mitochondrial complex III, inducing neurodegeneration characterized by psychomotor retardation or encephalopathy (59, 60). ATP5D is a subunit of ATP synthase, and its knockdown can lead to reduction in the ATP synthase level (61–63). COX6B1 is a subunit of COX complex, and is present in various types of cells, such as HeLa cells and yeast (64). In addition, recent research has reported that COX6B1 dysregulation could have significant effect on the COX functions, possibly resulting into the development of hydrocephalus, cerebromyopathy and other disorders (65, 66). The ATP5O expression has been reported to play critical roles in diagnosing and prognosing gastric cancer, and the analysis based on NextBio database reveals a downregulation of ATP5O expression in ccRCC (67, 68). ATP5H serves as an essential part of mitochondria responsible for energy production in eukaryotes; therefore, it is reasonable to expect dysregulation of ATP synthase expression in cancer cells would affect tumoral metabolism (69). The NDUFA6 protein is a subunit of NADH dehydrogenase (ubiquinone), the largest of five electron transport chain complexes localized within the internal membrane of mitochondria.

It has been reported that binding between the SARS-CoV-2 and ACE2 receptor could reduce the angiotensin 1-7 level, and induce vasoconstrictive, pro-inflammatory, and pro-coagulant effects, potentially resulting into vascular lesions in the placenta and preeclampsia (46, 70–72). Based on these data, it is of urgent need to perform mechanistic studies to better understand the infection of COVID-19 associated with preeclampsia. GO and KEGG analysis was performed in this study for identifying the connection between preeclampsia and COVID-19. clusterProfiler was used for GO analysis of BPs, CCs, and MFs. The MFs for these common DEGs primarily showed enrichment in the mitochondrial protein-containing complexes, inner membranes of mitochondria, and mitochondrial inner membrane protein complexes. The pathological and biochemical effects induced by COVID-19 could result into an acute inflammatory state (73). Such inflammatory conditions might have associations with hypermetabolic status, e.g., hyperglycemia, and with cellular dysregulation, e.g., mitochondrial dysfunction, considering their involvement in cellular functions and metabolic pathways (74, 75). Therefore, it is essential to analyze the roles played by cellular apparatus and molecules that have direct links with oxidative-stress regulations (73, 76). Identification of significant GO and molecular pathways could help us better understand the mechanism by which preeclampsia increases the COVID-19 mortality rate.

The links between TFs, miRNA, and common DEGs were then investigated. TFs are critical cellular expression-controlling factors. The activities of TFs dictate the cellular functions and responses to environments (77). TFs are also critical cancer-cell stemness enablers, providing support for maintenance and functions of cancer stem cells which are deemed as sources for tumoral development and metastasis, as well as drug resistance (78). In this study, ILF3, HTT, AR, MYC, RARA, IRF4, RAD21, JUN, SREBF1, and JUND were ranked and identified as the top 10 TFs based on the P-values. miRNAs could serve as both tumor suppressors and oncogenes depending on cellular conditions. miRNA could function as a down-regulator of mRNAs by competitively forming base-pairs with them (79). miRNA dysregulation has been reported to affect cancer phenotypes, including proliferative signaling activation, growth suppression evasion, apoptosis suppression, enhanced cellular invasion and metastasis, and increase of angiogenic activities (80). Hsa-miR-126-3p, hsa-miR-32-3p, hsa-miR-192-5p, hsa-miR-215-5p, hsa-miR-380-3p, hsa-miR-9-3p, hsa-miR-4666a-3p, hsa-miR-381-3p, hsa-miR-34a-5p, and hsa-miR-5692b were among the ten most significant miRNAs.

Furthermore, gene-disease relationship analysis was performed for the identification of common DEGs linked to diseases. It was revealed by the analysis that common DEGs are associated with various types of diseases including HIV and COVID-19, including Renal fibrosis, Ureteral obstruction, Colonic Neoplasms, Idiopathic Nephrotic Syndrome, IGA Glomerulonephritis, Burkitt Lymphoma, Medullary Neoplasms, Neuroblastoma, Congenital aneurysm of ascending aorta, and Mitochondrial Diseases. It has been suggested by recent studies that COVID-19 patients with cancer are more likely to develop severe symptoms and even suffer high mortality than non-cancer patients (81–86). COVID-19 is also shown to have a significant connection with neoplasms, such as gastrointestinal, colonic and prostatic neoplasm (83, 87, 88). These results are in consistency with our finding. More and more evidences suggest worse clinical outcomes in cancer patients with COVID-19 as opposed to non-cancer patients (89–93).

It is a high priority to develop effective and safe drugs for preeclampsia-affected COVID-19 patients. Multiple molecules and drugs have been identified in this study that might have therapeutic potential for COVID-19 patients affected with preeclampsia, including valproic acid, verteporfin, 7646-79-9, astemizole, vinblastine, colchicine, doxorubicin, ambroxol, phenobarbital, and copper sulfate. Valproic acid as well as its amidic derivatives (valnoctamide and valpromide) have long been demonstrated as an effective antiviral by inhibiting *in vitro* infection of a wide range of viruses (94, 95). Valproic acid may decrease the SARS-CoV-2 viral load, thus reducing infection risk by targeting the ACE2 receptor and transmembrane serine protease 2 (96). Verteporfin and protoporphyrin IX (PpIX), both approved by FDA, could inhibit the SARS-CoV-2-induced cytopathic effects, both at a low EC50 level (nanomolar) (97). Astemizole could also suppress the invasion of SARS-COV-2 Spike pseudovirus by binding with its ACE2 receptor (98). Colchicine can induce anti-inflammatory effects, and thus contribute to the mitigation of cytokine storm by acting on NLRP3 and inhibiting activities of IL-1β, IL-6, and IL-18 (99). Doxorubicin has reported efficacy against viral proteases in SARS-CoV-2 (100), while Ambroxol, known for its mucolytic effects in treating respiratory disorders, may show promise against SARS-CoV-2 by inducing autophagy (101).

Our study presents certain limitations. Firstly, while we identified potential drugs, the absence of clear targets necessitates further in-depth exploration into their specific mechanisms in future research. Secondly, our data did not include patients simultaneously affected by both COVID-19 and preeclampsia. Analyzing data with such dual conditions holds substantial implications. This analysis facilitates the identification of shared genetic features and molecular mechanisms between preeclampsia and COVID-19, shedding light on their underlying connections. Moreover, the discovery of common gene expression changes in both diseases opens avenues for potential novel biomarkers, contributing to the development of diagnostic or prognostic indicators. As pandemic restrictions ease, the increasing opportunities to collect specimens with these coexisting conditions will fuel continued in-depth research in the future.

In summary, our study may provide a new line of research in identifying the crosstalk between COVID-19 and Preeclampsia. First, we used GEO databases to identify hub genes which could contribute to the occurrence and development of COVID-19 and preeclampsia. Secondly, the interactions between CVOID-19 and preeclampsia were identified, thus shedding novel light on the molecular mechanisms that underlie the COVID-19 infection and preeclampsia. Finally, ten candidate drugs, which could potentially act as therapeutic biomarkers for COVID-19 and preeclampsia, were identified.

## Conclusions

In summary, this study uncovered shared molecular pathways and hub genes between COVID-19 and preeclampsia. The identified hub genes (MRPL11, MRPS12, UQCRH, ATP5I, UQCRQ, ATP5D, COX6B1, ATP5O, ATP5H, NDUFA6) exhibited distinct expression patterns validated through RT-qPCR. The findings provide crucial insights for potential therapeutic targets and further understanding of the molecular interplay between these conditions.

## Data availability statement

The original codes used for the analyses presented in the study are publicly available. This data can be found here: https://github.com/Liujunxiu97/Covid19-and-Preeclampsia.

## Ethics statement

The studies involving humans were approved by the affiliated hospital of Qingdao university. The studies were conducted in accordance with the local legislation and institutional requirements. The participants provided their written informed consent to participate in this study. The participants gave their consent to publish the study.

## Author contributions

YC, LX, and ML designed the project. YC, JW, MS and WX developed the experiments. YC, and LX analyzed the data. YC and LX wrote the paper. All authors contributed to the article and approved the submitted version.

## Supplementary material

Supplementary Figure 1 Identification of top 10 potential drugs. Drug names, P-values, molecular formulas, and two-dimensional structures are shown.

Supplementary Figure 2 ROC analysis of the hub genes in the COVID-19 dataset.

Supplementary Figure 3 ROC analysis of the hub genes in the preeclampsia dataset.

Supplementary Figure 4 The gene-disease association network represents diseases associated with common DEGs. The red squares represent the top 10 diseases with lowest P values. Other color circles represent common DEGs correlated with diseases.

Supplementary Table 1 qPCR primer sequences.

Supplementary Table 2 COVID-19 differentially expressed genes.

Supplementary Table 3 Preeclampsia differentially expressed genes.

Supplementary Table 4 Common DEGs between COVID-19 and preeclampsia.

Supplementary Table 5 The TFs and their interactions with DEGs.

Supplementary Table 6 The microRNAs and their interactions with DEGs.

Supplementary Table 7 The potential drugs for the treatment of COVID-19 and preeclampsia.

Supplementary Table 8 Regulatory interaction between common DEGs and diseases.
