# Supplementary figures and images for: Gene crosstalk between COVID-19 and preeclampsia revealed by blood transcriptome analysis
Source: Front Immunol. 2024 Jan 8;14:1243450. doi: 10.3389/fimmu.2023.1243450 (PMC10800816; doi:10.3389/fimmu.2023.1243450)

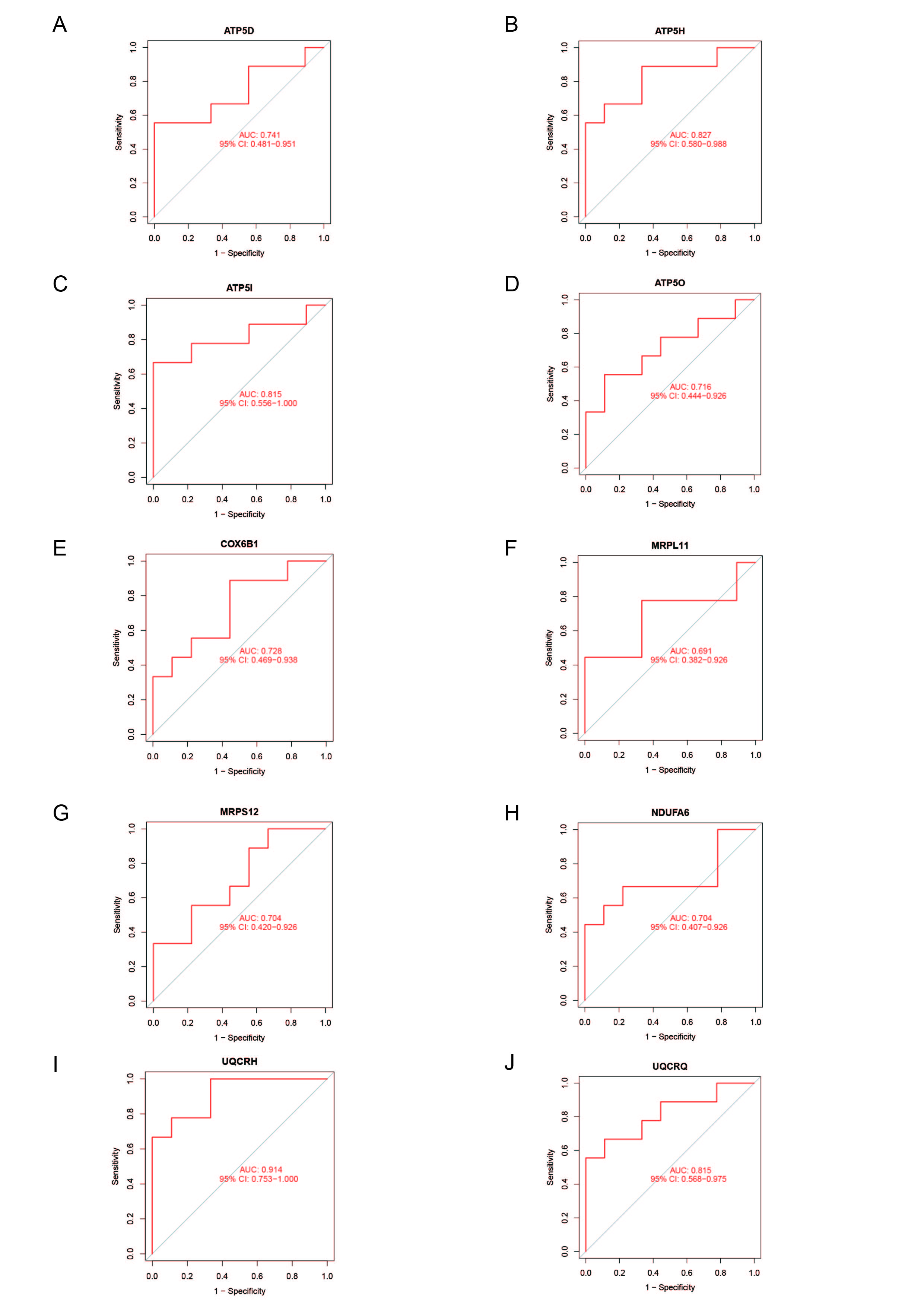

Supplement: Supplementary Figure 1 — Identification of top 10 potential drugs. Drug names, P-values, molecular formulas, and two-dimensional structures are shown. [file Image_1.jpeg]

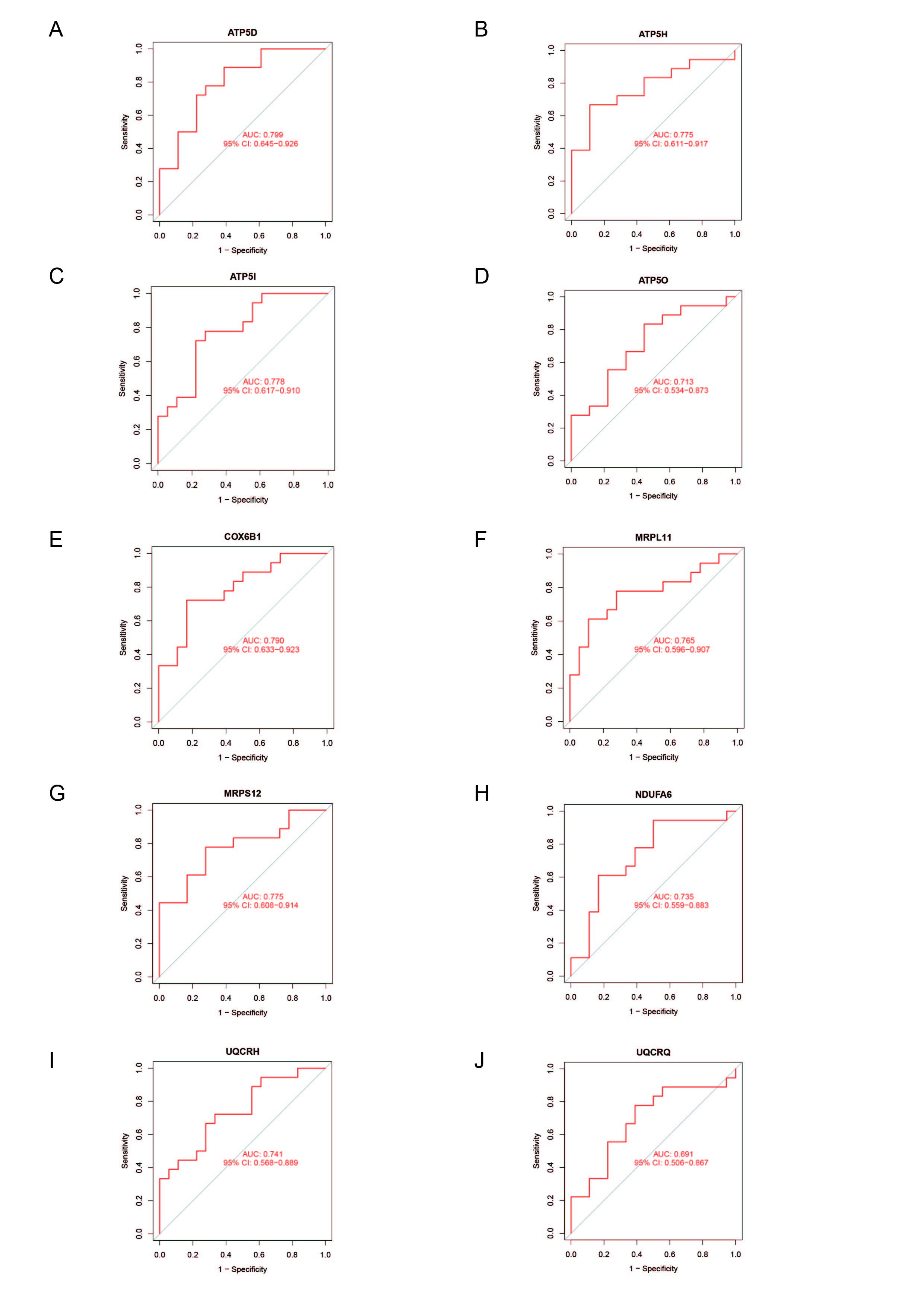

Supplement: Supplementary Figure 2 — ROC analysis of the hub genes in the COVID-19 dataset. [file Image_2.jpeg]

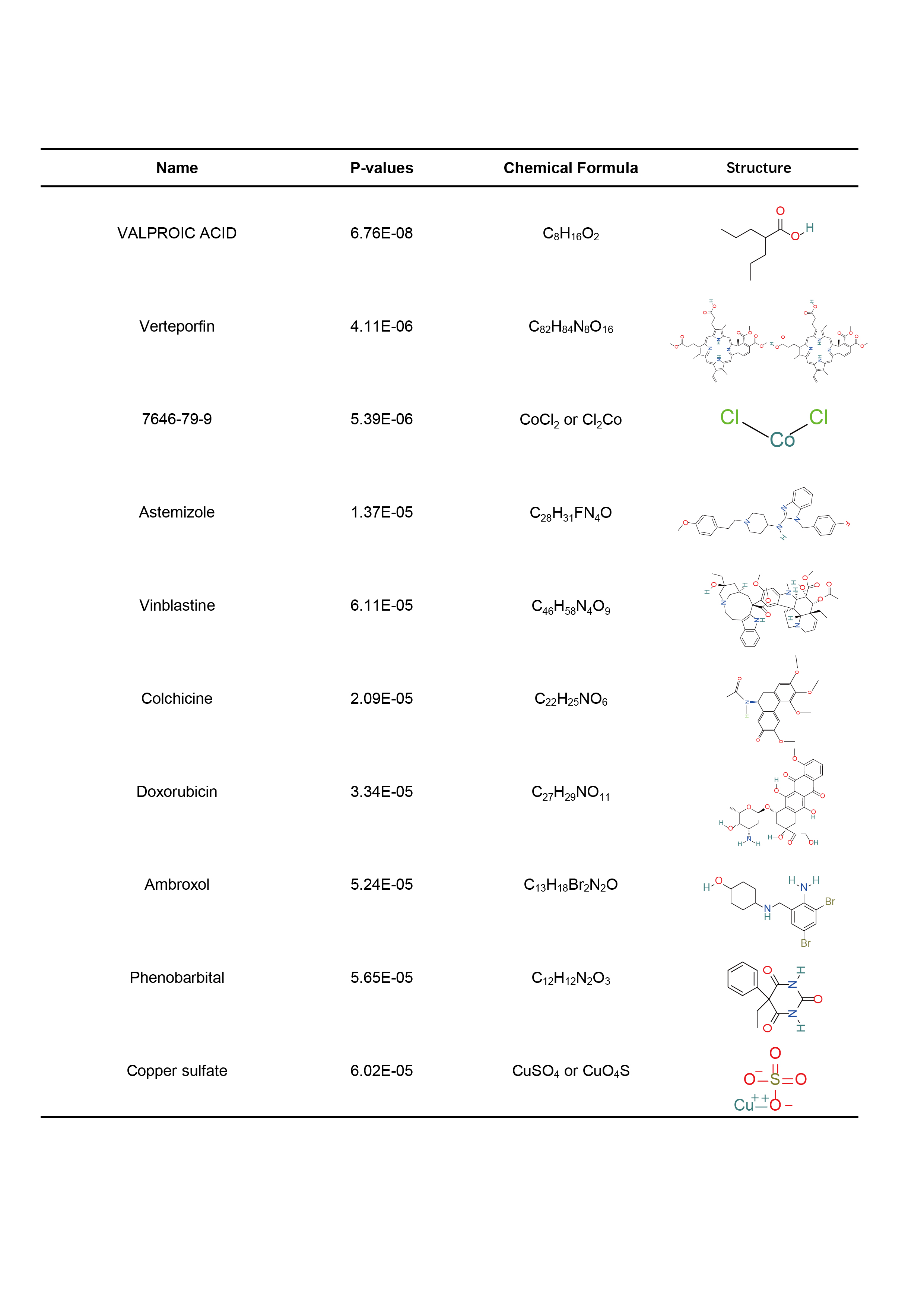

Supplement: Supplementary Figure 3 — ROC analysis of the hub genes in the preeclampsia dataset. [file Image_3.jpeg]

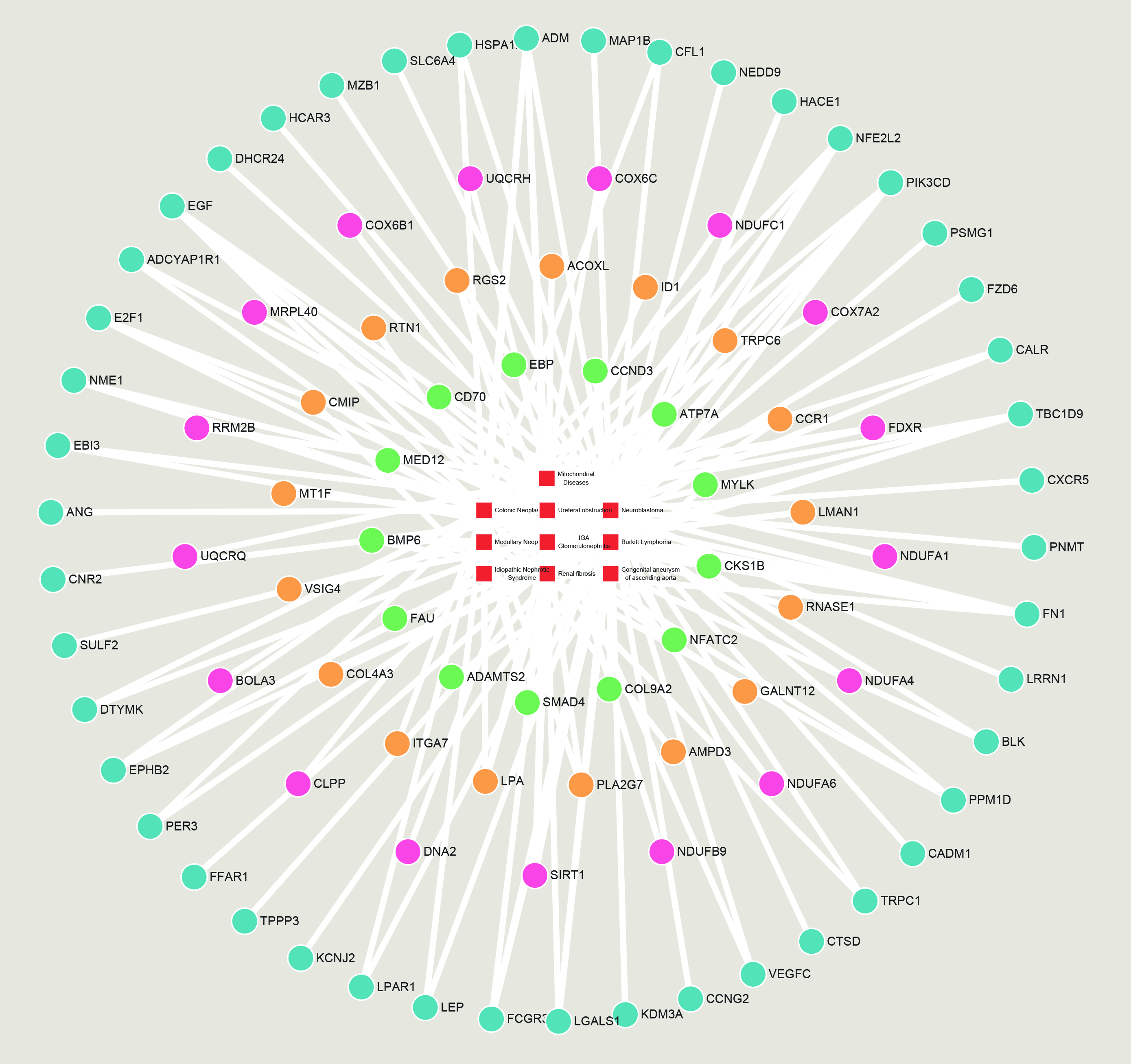

Supplement: Supplementary Figure 4 — The gene-disease association network represents diseases associated with common DEGs. The red squares represent the top 10 diseases with lowest P values. Other color circles represent common DEGs correlated with diseases. [file Image_4.jpeg]
